# Association between Diet, Physical Activity and Nutritional Status of Male Border Guard Officers

**DOI:** 10.3390/ijerph19095305

**Published:** 2022-04-27

**Authors:** Anna Anyżewska, Roman Łakomy, Tomasz Lepionka, Ewelina Maculewicz, Ewa Szarska, Andrzej Tomczak, Izabela Bolczyk, Jerzy Bertrandt

**Affiliations:** 1 University of Economics and Human Sciences in Warsaw, Okopowa 59, 01-043 Warsaw, Poland; 2 Military Institute of Hygiene and Epidemiology, 01-163 Warsaw, Poland; roman.lakomy@interia.pl (R.Ł.); tomasz.lepionka@wihe.pl (T.L.); eszarska@gmail.com (E.S.); 3 Faculty of Physical Education, Józef Pilsudski University of Physical Education in Warsaw, 00-809 Warsaw, Poland; ewelina.maculewicz@awf.edu.pl; 4 Independent Researcher, 02-348 Warsaw, Poland; biuro.at@onet.pl; 5 The Polish Border Guard Headquarters, 00-514 Warsaw, Poland; ibipolo@gmail.com; 6 Faculty of Economic Sciences, John Paul II University of Applied Sciences in Biala Podlaska, Sidorska 95/97, 21-500 Biała Podlaska, Poland; jwbertrandt@gmail.com

**Keywords:** diet, nutrition, physical activity, nutritional status, body mass index, fat mass index, bone mineral density, border guard officers

## Abstract

The main factors that determine the effectiveness and reliability of duties and tasks performed by border guard officers, are very good health and maintaining a high level of psychophysical fitness that depend mainly on adequate diet and physical activity and thus, nutritional status. The aim of the study was to verify the correlations between dietary habits, physical activity level and selected nutritional status indicators. One hundred and sixty-nine male border guard officers participated in the study. A 61-item food frequency questionnaire (FFQ) was used to assess dietary habits and a long-form International Physical Activity Questionnaire (IPAQ) was used to assess physical activity. Fat mass was determined by bioelectrical impedance analysis (BIA) and bone calcification was assessed by the dual energy X-ray absorptiometry (DXA). Many correlations between dietary habits, as well as the physical activity of officers and body mass index (BMI), fat mass index (FMI) and visceral fat level (VFL) were found, while bone mineral density (BMD T-score) negatively correlated only with two food groups and 6 out of 61 products but did not correlate with physical activity. The results also confirmed many poor dietary habits and abnormalities in nutritional status. Thus, there is a need for nutritional education and further monitoring of health-related behaviors, as well as monitoring the nutritional status of border guard officers.

## 1. Introduction

Very good health and maintaining high level of psychophysical fitness are the main factors that determine the effectiveness and reliability of duties and tasks performed by Border Guard officers. The main tasks include border protection and the control of border traffic [1]. Some of the border guard’s duties are performed in diverse geographical conditions, such as the sea area or mountains, thus maintaining a healthy condition and psychophysical fitness are crucial. Changing circumstances, i.e., during the deployment period, may lead to modification of dietary habits and exercise routine, for example with a negative effect on body composition and physical performance [2]. Thus, it is very important to maintain good nutritional status which mainly depends on diet and physical activity.

Optimizing nutrition strategies to support health and performance is important, especially for physically active people [3] as well as tactical personnel [4]. The recommendations, for example position stands developed by the International Society of Sports Nutrition, such as nutrient timing [5], protein and exercise [6] can be also applied as some of the nutrition recommendations for uniformed forces. Nevertheless, the basic principles of healthy diet and implementing a balance and diverse diet with high nutritional density are crucial, especially considering the poor dietary habits observed in these populations [4,7,8,9,10]. Diet quality, eating styles, and macronutrient composition influence body composition [11]. Too low an energy intake can lead to weight loss, especially a decrease in muscle mass, or decrease in bone density, which may negatively affect psychophysical performance, prolong recovery time and increase injury risk [2,12]. On the other hand, too high an energy intake may cause weight gain, and thus indirectly increase the risk of obesity that might result in difficulties in fulfilling service tasks. These abnormalities might be also the reason for early service eliminations because of health concerns.

The second factor that significantly affects body composition is physical activity. It not only leads to increasing physical performance and muscle mass, but also, as the most variable component of daily energy expenditure, determines energy balance [13]. Lifelong exercise delays the onset of 40 chronic diseases, such as coronary (ischemic) heart diseases, hypertension, obesity, insulin resistance, metabolic syndrome, osteoporosis, depression, and anxiety [14]. The evidence for the notion of “exercise is medicine” is strong, and physical activity has been used in both the prevention and treatment strategies for various diseases [15]. It is also an interaction between physical activity and other factors, for example diet and genetics that increases disease risk factors.

Since physical inactivity is considered as the biggest public health problem of the 21st century [16], the growing trend of people with overweight and obesity is also observed in the general population [17,18], as well as among uniformed service officers [19,20,21,22,23,24,25]. According to the last study, the prevalence of overweight and obesity in the Polish Army Forces (50% and 17% of men) [10] is similar to that in the general population of Poland (52% and 16% of men) [18]. Border guard officers, like other uniformed services, attend physical education classes and are obligated to complete annual physical fitness test [26,27]. Nevertheless, it has been observed that not all of border guard officers attend sport classes [28,29,30]. It has been shown that both occupational and leisure time physical activity are associated with body composition in police officers [31], thus it is necessary to assess the level of physical activity including both physical activity during work and physical activity as sport and leisure time.

Proper diet, regular physical activity, and thus nutritional status are extremely important for uniformed forces, affecting their physical fitness and their suitability for service. However, the literature on these associations between lifestyle factors and body composition among tactical personnel is relatively scarce, especially there has been a lack of research carried out among border guard officers. Therefore, the aim of the study was to verify the correlations between dietary habits, physical activity level and selected nutritional status indicators: body mass index (BMI), fat mass index (FMI), visceral fat level (VFL), bone mineral density (BMD T-score) and muscle mass index (MMI) in border guard officers from Poland.

## 2. Materials and Methods

The study was carried out with the participation of 169 male border guard officers from Poland. Informed consent was obtained from all subjects involved in the study. The study was conducted in accordance with the Declaration of Helsinki and approved by the Ethics Committee of the Military Institute of Hygiene and Epidemiology (1/XXI/2016).

### 2.1. Eating Meals, Food Consumption Frequency Assessment and Dietary Patterns

A two-part questionnaire was used to assess diet. The first part contained questions about the regularity of eating five meals with three possible answers to choose: every day, not every day, never. The second part was the evaluated 61-item food frequency questionnaire (FFQ) [32], slightly modified by adding two new categories of answers in this questionnaire, as in an earlier study [9].

Border guard officers were asked, included in the FFQ, how often they had consumed 61 food products in the past 12 months. For each product they could choose one of the eight answers regarding food consumption frequency: 1—never or almost never, 2—once a quarter or less often, 3—once a month or less often, 4—a few times a month, 5—once a week, 6—several times a week, 7—every day, 8—several times a day.

Reported frequencies were calculated into daily frequencies (time/day), as follows: never or almost never—0.003 (1/365), once a quarter or less often—0.01 (4/365), once a month or less often—0.03 (1/30), a few times a month—0.08 (2.5/30), once a week—0.14 (1/7), several times a week—0.57 (4/7), every day—1, several times a day—2. Converted frequencies were also summed within the main food groups, that were identified as:

1. Fruits, vegetables, and potatoes.
2. Seeds of legumes.
3. Cereal products.
4. Dairy products and eggs.
5. Meat products and fish.
6. Fats, nuts, and grains
7. Sweets and snacks.
8. Soft drinks.
9. Alcoholic beverages.

Converted frequencies of the main food groups were used to obtain dietary patterns.

### 2.2. Physical Activity Assessment

A long-form International Physical Activity Questionnaire (IPAQ) was used to assess physical activity level [33]. Officers were asked to answer 27 questions about physical activity during work/job, transportation, homework, house maintenance, and caring for family, recreation, sport, and leisure-time, as well as time spent sitting. Then results were calculated as metabolic equivalent (MET-minutes/week) according to the scoring protocol [34].

### 2.3. Nutritional Status Assessment

The TANITA HR-001 stadiometer (Tanita Corporation, Tokyo, Japan) was used to measure height. The head was aligned in the Frankfort horizontal plane [35]. Body weight and body composition (fat mass, visceral fat level, muscle mass) were evaluated using the TANITA MC-780 analyzer (Tanita Corporation, Tokyo, Japan). All measurements were performed in accordance with the procedures from the instruction manuals. Three additional indicators were calculated: body mass index (BMI), fat mass index (FMI) and muscle mass index (MMI):

BMI = body weight/height^2^ [kg/m^2^],

FMI = fat mass/height^2^ [kg/m^2^],

MMI = muscle mass/ height^2^ [kg/m^2^].

The scale of BMI classification reported by the World Health Organization (WHO) [36] and the scale of FMI described by Kelly et al. [37] were accepted.

Bone mineral density (BMD) was measured in the forearm bone of the nondominant hand, using Dual Energy X-ray Absorptiometry (DEXA) densitometric method, with an EXA 3000 analyzer (OsteoSys, Seoul, Korea). The results were interpreted in accordance with the WHO standards for BMD T-score: osteoporosis: BMD T-score ≤ −2.5, osteopenia: −2.5 < BMD T-score ≤ −1.0, standard: BMD T-score > −1.0 [38].

### 2.4. Statistical Analysis

The PS IMAGO PRO (IBM SPSS Statistics, Armonk, NY, USA) program was used for all statistical analyses. Shapiro–Wilk test was used to verify the compatibility of variable distribution with normal distribution. Due to noncompliance of analyzed variables with normal distribution, Spearman’s correlation was conducted to assess the associations among dietary habits, physical activity and five nutritional status indicators (BMI, FMI, VFL, BMD T-score, and MMI). Two dietary patterns were identified using the K-means cluster analysis. Input variables were converted to standardized scores. Logistic regression was used to assess the relationship between excess fat mass and potential risk factors. For all analysis the significant level of α = 0.05 was assumed.

## 3. Results

The study was conducted among 169 male border guard officers aged 37 ± 6 years. The average length of service was 13 ± 7 years. About 1/3 of the subjects lived in a city of over 100 thousand inhabitants (32%), 35% in a city of up to 100 thousand inhabitants, and the rest lived in the country (33%). Almost 3/4 of officers were higher educated (71%), while others were secondary educated (29%). Nearly 2/3 of subjects assessed the physical level of work as light (27%) or performed while sitting (38%). According to 30% of officers, their work was moderate, and for others it was hard (5%).

### 3.1. Eating Meals, Food Consumption Frequency and Dietary Patterns

The meals that were most often eaten every day were dinner (84%), breakfast (83%) and supper (66%) (Table 1). Lunch was eaten every day only by 30%, afternoon snack by 18%, and supper by 66% of officers.

Fruits (all types) were eaten every day by only 30% of subjects, out of which barely 3% ate them several times a day (Table S1). About half of officers (51%) ate fruits several times a week. The most common fruits consumed were apples and pears (eaten more than once a week by 61% of subjects), and bananas (eaten more than once a week by 43% of subjects), while avocado, olives and other tropical fruits (not including kiwi fruit and citrus) were the least often consumed. Vegetables (all types) were eaten every day by only 32% of officers, including 3% of them who ate these products several times a day. Almost half of subjects (49%) ate vegetables several times a week. Tomatoes were the most often consumed vegetable (eaten more than once a week by 73% of officers), while leafy green vegetables and crucifers were the least often consumed (eaten more than once a week by 31% and 33 % of officers). So-called dark bread (wholemeal or with grains) was eaten every day by only 26%, while so-called white bread by 33%. Most of the officers consumed all dairy products, as well as eggs, less than daily. Meat products, especially high-quality cold-cuts and sausages were much more popular than fish—both lean and oily fish were eaten less than once a week by most of the officers (69% and 75%). Although nuts were more popular than grains, only 21% and 10% of subjects ate these products more than once a week. Sweets were more popular than salty snacks. More than half of officers (53%) never or almost never consumed energy drinks, while sweetened sodas such as beer were consumed more than once a week by 23% and 28% of subjects. Two major dietary patterns were identified using the K-means cluster analysis. The first one Dietary pattern 1—was a more healthy and more varied diet, while Dietary pattern 2 was a less healthy and less varied diet. Barely 24% of border guard officers were classified in the more healthy diet group (Dietary pattern 1). The differences between these two dietary patterns are described in Table 2.

### 3.2. Physical Activity

Based on the IPAQ, the average total physical activity equaled 17,255 ± 14,152 (median: 13,692) MET-minutes per week (Table 3). The largest part of physical activity was job-related physical activity (39%). Housework, house maintenance, caring for family accounted for 22%, transportation for 20%, and recreation, sport and leisure-time physical activity for 19% of the total value of physical activity. Taking into consideration the intensity level, walking counted for 38%, moderate physical activity for 36%, and intensive physical activity for 26% of the total value of physical activity. According to the IPAQ classification, 93% officers were characterized by a high and 7% by a moderate level of physical activity. The mean time spent in the sitting position on weekdays was 4.8 ± 2.8 (median: 4.0) hours a day, and on weekends it was 3.9 ± 2.3 (median: 4.0) hours a day, however the result values ranged from 0.5 up to 14.5 h a day.

### 3.3. Nutritional Status

Nutritional status indicators varied among the examined border guard officers (Table 4). Body weight ranged from 62.5 to 114.9 kg and height ranged from 165.6 to 199.3 cm. According to the BMI classification, normal weight was observed in only 32% of officers, while 67% officers were overweight (53%) or obese (14%). However, normal fat was found in 54% of officers, and excess fat in 39%. Additionally, in the group with BMI higher than 25 kg/m^2^, 58% of the officers were normal fat, and only 42% were classified as excess fat. Almost all of participants (96%) had a healthy level of visceral fat, according to the Tanita classification. Sufficient bone mineral density was observed in 87% of the subjects, osteopenia—12%, and osteoporosis—1%.

Since there were no associations between age and BMI, FMI, BMD (t-score) and MMI, age was positively correlated with VFL (Table 5). The nutritional status indices were correlated with each other.

### 3.4. Associations among Diet, Physical Activity and Nutritional Status

Twelve negative correlations between the main food groups consumption frequency and BMI (fruits, vegetables, and potatoes; seeds of legumes; dairy products and eggs; fats, nuts, and grains), FMI (fruits, vegetables, and potatoes; seeds of legumes; dairy products and eggs) VFL (fruits, vegetables, and potatoes; seeds of legumes; dairy products and eggs) and BMDT-score (fruits, vegetables, and potatoes; dairy products and eggs) were found (Table S2). Out of the selected 61 products negative correlations were observed between BMI and 18 products (fruits together—all type; other tropical fruits; bananas; sweet fruit preserves and candied fruits; vegetables—all types; yellow–orange vegetables; leafy green vegetables; fresh seeds of legumes and canned ones; potatoes in various forms; unrefined groats coarse; refined cereal grain; ready-to-eat breakfast cereal products; milk and milk drinks; butter; nuts; grains; honey; vegetable juices and vegetable-fruit ones). FMI negatively correlated with almost all of products from the first group, including fruits, vegetables, and potatoes (14 of 18), and with seeds of legumes, unrefined groats coarse; refined cereal grain; ready-to-eat breakfast cereal products; milk and milk drinks; lean fish; nuts; grains; honey; vegetable juices and vegetable-fruit ones. Positive correlation was observed only between FMI and sweetened sodas such as Fanta, Coca-Cola, Mirinda, Sprite. VFL negatively correlated with 20 out of 61 analyzed foods. The BMD T-score negatively correlated with six products (yellow–orange vegetables; tomatoes; potatoes; cottage cheese; eggs; honey).

It was found that officers with more healthy dietary habits (dietary pattern 1) had significantly lower BMI (*p* = 0.001), FMI (*p* < 0.001) and VFL (*p* < 0.001) than officers from the less healthy diet group (Table 6).

No associations between the total amount of physical activity and BMI, FMI, VFL, and BMD T-score were observed (Table 7). However, correlations were found between several categories of physical activity and BMI, FMI and VFL. Recreation, sport and leisure-time physical activity negatively correlated with BMI (Rho = −0.182; *p* = 0.018), FMI (Rho = −0.277; *p* < 0.001) and VFL (Rho = −0.228; *p* = 0.003), while waking negatively correlated only with BMI (Rho = −0.156; *p* = 0,043). Time spent sitting at the weekend positively correlated with FMI (Rho = 0.198; *p* = 0.014) and VFL (Rho = 0.172; *p* = 0.034). Only one correlation was found between muscle mass expressed as MMI and walking (Rho = −0.186; *p* = 0.015).

It was found that both dietary pattern (OR = 2.98) and physical activity: sport and recreation (OR = 0.57) are associated with the risk of excess fat mass (Table 8).

## 4. Discussion

Our results confirm that there are some relationships between diet, physical activity and nutritional status indicators assessing body mass, fat mass, visceral fat mass and bone mineral density among border guard officers. Many irregularities in dietary habits (i.e., insufficient meals inadequate consumption of some food groups) as well as in nutritional status were also observed.

Poor eating habits resulted in abnormalities in officers’ nutritional status. The strongest correlations (negative) were found between BMI, FMI and VFL, and nuts, fruit and vegetables, and dairy products frequency consumption. The frequency of the consumption of these products was not compatible with the current recommendations. Barely 1/3 of officers ate fruits and vegetables daily, but only 3% ate these products a few times a day. Our finding are worrying and in line with the general tendency of decreasing fruit and vegetable consumption in past years, that has been observed in household budget surveys by Statistics Poland [39]. Between 2000 and 2018, the average monthly consumption per capita of fruit decreased from 4.1 kg to 3.7 kg, and the average monthly consumption per capita of vegetables decreased from 13.3 to 7.9 kg. Similar dietary mistakes, i.e., consuming less than the recommended 4–5 meals a day, insufficient consumption of fruit, vegetables, nuts, diary, were also observed in the general population in Poland [40], as well as among soldiers [7,9] and other physically active groups, i.e., athletes [41]. Poor dietary habits may result in a worse body composition and can lead to obesity [11]. In a recent study, Gaździńska et al. observed that consumption of sweetened beverages was higher in soldiers with BMI ≥ 30 as compared to normal weight soldiers, while there were no differences in the number of meals during the day, snacking between meals, or fast-food consumption [10]. However, most of the surveyed declared eating sweets (80%) and fast-food (69%), and only 33% of soldiers were classified with normal weight. The authors showed that the risk of obesity increases with the age of 40, but is not limited to this, due to over consumption of food in stressful situations and lower physical activity.

The level of physical activity in almost all of the border guard officers was high (93%), similar to the earlier research among soldiers [9]. No correlations between total physical activity and BMI, FMI VFL, BMD-T-score, and MMI were observed. MMI was associated only with walking (negative correlation) while only physical activity during recreation, sport, and leisure-time negatively correlated with BMI and FMI. Similar relationships between LTPA and body composition were also observed in police officers [31,42]. Police officers with higher physical activity level had lower fat mass. It was also reported that police officers who were physically active and had a low level of body fat had better reaction times [43]. In another study it was observed that high level of body fat was associated with low level of physical performance [44]. In our study, time spent sitting positively correlated with FMI, and this was also observed by other authors [45,46].

During physical education classes border guard officers attend classes such as swimming and water rescue, water sports, martial arts, and training of the use of firearms. In previous studies it was shown that in a group of 55 female border guard officers almost 2/3 practiced sports (5% competitive, and 69% recreational), but 26% did not perform any leisure-time physical activity [28]. In another study, in another group of 53 male and female border guard officers only 36% were physically active in their spare time [30]. However, in a different study with a larger surveyed group (121 female and 338 male) the percentage of not performing any leisure-time physical activity was lower—7% of female and 4% of male [29]. According to the IPAQ classification the level of physical activity in border guard officers from their own research was higher than in the last study performed by Łyżwiński (93% with the high and 7% with the moderate level of physical activity vs. 58% with the high, 36% with the moderate, and 6% with the low level of physical activity) [47]

According to the BMI, excessive body mass was found in 67%, while based on FMI, excessive amount of fat mass was found in 39% of border guard officers. Our results are consistent with a recent study describing the ratio of overweight or obesity in 69% men from Poland aged 18–64 [18]. Border guard officers were more likely to have excess fat mass than soldiers from Poland [9] (39% vs. 19%), while the average BMI value was more similar (26.6 vs. 25.6 kg/m^2^). High prevalence of uniformed force officers with overweight or obesity was also observed in other studies, i.e., among police officers [21,48], soldiers [9,19,20,22,23,24,25], and firefighters [49]. However, it must be highlighted that using only the BMI classification to verify overweight or obesity, especially in physically active groups may be incorrect due to usual extensive muscle mass [50]. The authors found that major discrepancy exists between obesity according to BMI (about 25%) and being diagnosed for obesity with the ICD code (12.5% of diagnosed for obesity by BMI). In our study similar differences were also observed. The average BMI was higher than the reference value for normal weight, while the average FMI was adequate. Out of 67% of the officers classified with BMI higher than 25 kg/m^2^, excessive fat mass (based on FMI) was found only in less than half of them (42%). It is strongly recommended to be particularly careful when interpreting BMI values in adults with increased physical activity [51]. Moreover, some authors recommend that the optimal cut off point for interpreting obesity in active duty service members is BMI of 29 kg/m^2^ in men and BMI of 26 kg/m^2^ in women [52]. It means that BMI 25–29 kg/m^2^ may be not useful for clearly discriminating between lean and fat mass in physically actives, especially active-duty service members [22,50]. In one of the recent studies, there was an attempt to assess adiposity in the U.S. The military based a combination of BMI + circumference-based equations, however it resulted in poor sensitivity [25].

The main limitation of our study is the relatively small group (169 officers). We conducted our research only among men, so future studies should include both men and women. The other limitation is using FFQ to assess diet. Although it is not an ideal method, it has been widely used in various studies. Some of the obtained Spearman’s correlations might be difficult to interpret based on only FFQ without portions of consumed foods. Thus, in future studies comparing nutritional status with energy intake would be an additional benefit. The other limitation is using only IPAQ to assess the physical activity level. For future studies, it is recommended to use an accelerometer and add more detailed questions on the type of training (i.e., strength or endurance training). Energy as well as macronutrient intake (protein, fat and carbohydrate) and a detailed description of physical activity could be used for more detailed analyses, i.e., to assess associations between muscle mass and dietary intakes and physical activity among uniformed service officers.

## 5. Conclusions

Our study confirmed correlations between diet, physical activity and body mass index, fat mass index, visceral fat level, and bone mineral density in male border guard officers. Higher body mass and fat mass were correlated with poorer dietary habits, i.e., low consumption of fruit, vegetables, dairy, nuts, grains, as well as with lower leisure-time physical activity and longer time spent sitting during the day.

There is a strong need for further monitoring of health-related behaviors among border guard officers due to the many dietary mistakes and abnormalities in nutritional status that were observed. It is also necessary to provide nutritional education and encourage officers to follow dietary recommendations.

## Figures and Tables

**Table 1:** Frequency of eating meals by border guard officers.

Percentage of Answers (%)	Breakfast	Lunch	Dinner	Afternoon Snack	Supper
Every day	83	30	84	18	66
Not every day	14	36	16	27	26
Never	3	34	0	55	8

**Table 2:** The frequency of food consumption by dietary pattern groups.

Dietary Pattern/Groups of Products	Dietary Pattern 1		Dietary Pattern 2		*p*
	X ± SD	Me	X ± SD	Me	
Fruits, vegetables, and potatoes	8.21 ± 3.50	8.07	4.69 ± 2.14	4.63	<0.001 **
Seeds of legumes	0.32 ± 0.30	0.16	0.13 ± 0.16	0.11	<0.001 **
Cereal products	2.66 ± 0.99	2.79	1.49 ± 0.70	1.35	<0.001 **
Dairy products and eggs	3.29 ± 1.52	3.25	1.63 ± 0.92	1.64	<0.001 **
Meat products and fish	2.90 ± 1.27	2.93	1.61 ± 0.78	1.53	<0.001 **
Fats, nuts, and grains	2.69 ± 1.04	2.70	1.61 ± 0.88	1.54	<0.001 **
Sweets and snacks	2.35 ± 1.32	2.33	1.22 ± 0.87	1.09	<0.001 **
Non-alcoholic beverages	1.18 ± 0.83	1.03	0.46 ± 0.46	0.26	<0.001 **
Alcoholic beverages	0.51 ± 0.46	0.42	0.33 ± 0.33	0.24	0.041 *
Selected products					
Fruits, vegetables, and potatoes					
Fruits together—all types	0.89 ± 0.46	1.00	0.56 ± 0.32	0.57	<0.001 **
Stone fruits	0.41 ± 0.39	0.36	0.19 ± 0.22	0.08	<0.001 **
Kiwi fruit and citrus	0.49 ± 0.41	0.57	0.17 ± 0.24	0.08	<0.001 **
Other tropical fruits	0.20 ± 0.34	0.08	0.09 ± 0.15	0.03	0.001 **
Bananas	0.57 ± 0.44	0.57	0.29 ± 0.30	0.14	<0.001 **
Apples and pears	0.60 ± 0.43	0.57	0.38 ± 0.28	0.57	0.002 **
Avocado	0.06 ± 0.13	0.01	0.05 ± 0.12	0.01	0.185
Olives	0.09 ± 0.17	0.03	0.08 ± 0.16	0.03	0.787
Dried fruits	0.18 ± 0.29	0.08	0.11 ± 0.20	0.03	0.107
Sweet fruit preserves and candied fruits	0.18 ± 0.25	0.08	0.09 ± 0.18	0.03	0.014 *
Vegetables—all types	0.89 ± 0.41	1.00	0.58 ± 0.36	0.57	<0.001 **
Crucifers	0.39 ± 0.36	0.57	0.22 ± 0.23	0.14	0.002 **
Yellow–orange vegetables	0.56 ± 0.35	0.57	0.28 ± 0.22	0.14	<0.001 **
Green leafy vegetables	0.44 ± 0.27	0.57	0.21 ± 0.24	0.08	<0.001 **
Tomatoes	0.71 ± 0.37	0.57	0.48 ± 0.31	0.57	0.001 **
Vegetables: fresh cucumbers, squash, zucchini, pumpkin, eggplant	0.59 ± 0.36	0.57	0.33 ± 0.28	0.14	<0.001 **
Root vegetables and others	0.50 ± 0.28	0.57	0.27 ± 0.24	0.14	<0.001 **
Potatoes in various forms	0.58 ± 0.38	0.57	0.40 ± 0.30	0.57	0.005 **
Seeds of legumes					
Fresh seeds of legumes and canned ones	0.22 ± 0.22	0.08	0.08 ± 0.10	0.08	<0.001 **
Dry seeds of legumes	0.10 ± 0.12	0.08	0.05 ± 0.07	0.03	<0.001 **
Cereal products					
Wholemeal or with grains, so-called dark bread	0.75 ± 0.58	0.57	0.47 ± 0.40	0.57	0.006 **
Refined bread, so-called white bread	0.81 ± 0.57	0.79	0.54 ± 0.47	0.57	0.004 **
Unrefined groats coarse	0.39 ± 0.27	0.57	0.19 ± 0.22	0.08	<0.001 **
Refined cereal grain	0.39 ± 0.25	0.57	0.17 ± 0.19	0.08	<0.001 **
Ready-to-eat breakfast cereal products	0.32 ± 0.33	0.14	0.12 ± 0.20	0.03	<0.001 **
Dairy products and eggs					
Milk and milk drinks	0.82 ± 0.59	0.57	0.40 ± 0.36	0.57	<0.001 **
Sweetened milk drinks	0.45 ± 0.43	0.57	0.19 ± 0.24	0.08	<0.001 **
Cottage cheese	0.58 ± 0.29	0.57	0.29 ± 0.27	0.14	<0.001 **
Flavored cottage cheese	0.26 ± 0.32	0.08	0.08 ± 0.14	0.03	0.004 **
Cheese	0.63 ± 0.37	0.57	0.34 ± 0.31	0.14	<0.001 **
Eggs and egg dishes	0.54 ± 0.28	0.57	0.33 ± 0.26	0.14	<0.001 **
Meat products and fish					
Sausages. different types	0.68 ± 0.41	0.57	0.37 ± 0.29	0.57	<0.001 **
High-quality cold cuts	0.72 ± 0.31	0.57	0.47 ± 0.30	0.57	<0.001 **
Sausage products and offal	0.26 ± 0.30	0.14	0.11 ± 0.16	0.08	<0.001 **
Red meat	0.36 ± 0.40	0.14	0.17 ± 0.20	0.08	0.001 **
Poultry and rabbit	0.51 ± 0.27	0.57	0.31 ± 0.25	0.14	<0.001 **
Wild game meat	0.05 ± 0.12	0.01	0.02 ± 0.06	0.01	0.034 **
Lean fish	0.16 ± 0.18	0.14	0.08 ± 0.09	0.08	0.003 **
Oily fish	0.16 ± 0.20	0.08	0.08 ± 0.11	0.06	0.003 **
Fats, nuts, and grains					
Oil, all kinds	0.52 ± 0.33	0.57	0.32 ± 0.26	0.14	0.002 **
Butter, all types	0.81 ± 0.43	1.00	0.52 ± 0.47	0.57	<0.001 **
Margarine, all types	0.30 ± 0.50	0.08	0.21 ± 0.35	0.03	0.073
Cream, sweet or sour cream, for food or beverages	0.30 ± 0.29	0.14	0.16 ± 0.26	0.08	0.005 **
Other animal fats	0.08 ± 0.15	0.03	0.07 ± 0.16	0.03	0.532
Mayonnaise and dressings, i.e., salad dressings—all types	0.23 ± 0.38	0.08	0.11 ± 0.15	0.08	0.035 *
Nuts	0.28 ± 0.29	0.14	0.16 ± 0.22	0.08	0.049 *
Grains	0.21 ± 0.28	0.08	0.09 ± 0.17	0.03	0.010 **
Sweets and snacks					
Sugar to sweeten beverages	0.89 ± 0.80	1.00	0.43 ± 0.58	0.08	0.003 **
Honey to sweeten food and beverages	0.24 ± 0.30	0.08	0.15 ± 0.26	0.03	0.062
Chocolate, chocolate candies, and candy bars	0.48 ± 0.33	0.57	0.25 ± 0.30	0.08	<0.001 **
Non-chocolate candies	0.22 ± 0.41	0.08	0.08 ± 0.15	0.03	0.030 *
Biscuits and cakes	0.30 ± 0.29	0.14	0.16 ± 0.20	0.08	0.004 **
Ice cream and pudding	0.08 ± 0.09	0.08	0.07 ± 0.13	0.03	0.035 *
Salty snacks	0.18 ± 0.29	0.08	0.08 ± 0.12	0.03	0.146
Soft drinks					
Fruit juices and fruit nectars	0.47 ± 0.40	0.57	0.23 ± 0.27	0.08	<0.001 **
Vegetable juices and vegetable-fruit ones	0.35 ± 0.45	0.14	0.09 ± 0.15	0.03	<0.001 **
Energy drinks	0.12 ± 0.25	0.03	0.04 ± 0.20	0.00	<0.001 **
Sweetened sodas such as Fanta, Coca-Cola, Mirinda, Sprite	0.24 ± 0.29	0.08	0.10 ± 0.17	0.03	0.034 *
Alcoholic beverages					
Beer	0.34 ± 0.30	0.14	0.20 ± 0.24	0.08	0.002 **
Wine and drinks	0.10 ± 0.19	0.03	0.08 ± 0.12	0.03	0.804
Vodka and spirits	0.07 ± 0.12	0.03	0.06 ± 0.10	0.03	0.931

U Mann–Whitney Test * *p* < 0.05; ** *p* < 0.01. X—average; SD—standard deviation; Me—median.

**Table 3:** Physical Activity according to the long-form International Physical Activity Questionnaire (IPAQ).

Characteristics	X ± SD	Me	IRQ
Physical activity (MET-minutes/week):			
total	17,255 ± 14,152	13,692	6606–24,390
job-related physical activity	6803 ± 8469	3390	480–9900
transportation	3418 ± 3802	2133	495–5148
housework, house maintenance, caring for family	3793 ± 4398	2295	660–5040
recreation, sport and leisure-time physical activity	3241 ± 4560	1680	420–3919
walking	6548 ± 6672	3861	1663–8910
moderate	6232 ± 6144	4320	2160–8550
intensive	4475 ± 5737	2160	480–6400
Time spent sitting (h/d):			
weekdays	4.8 ± 2.8	4.0	3.0–6.0
weekend	3.9 ± 2.3	4.0	2.0–5.0

X—average; SD—standard deviation; Me—median; IRQ—interquartile range.

**Table 4:** Anthropometry and nutritional status.

Characteristics	X ± SD	Me
Height (cm)	179.3 ± 6.3	179.0
Weight (kg)	85.7 ± 11.4	84.4
BMI (kg/m^2^)	26.6 ± 3.2	26.2
FMI (kg/m^2^)	5.6 ± 1.9	5.4
MMI (kg/m^2^)	20.0 ± 1.4	19.9
	Percentage of classifications	
BMI (kg/m^2^)	Underweight < 18.5	1
	18.5 ≤ Norm < 25.0	32
	25.0 ≤ Overweight < 30.0	53
	Obesity > 30.0	14
FMI (kg/m^2^)	Fat deficit <3	7
	3 ≤ Normal fat ≤ 6	54
	Excess fat >6	39
VFL	Healthy level	96
	Excess level	4
BMD T-score	Osteoporosis ≤ −2.5	1
	−1.0 ≥ Osteopenia > −2.5	12
	Standard > −1.0	87

X—average; SD—standard deviation; Me—median; BMI—body mass index; FMI—fat mass index; MMI—muscle mass index; VFL—visceral fat level; BMD T-score—bone mineral density expressed in relation to a reference population in standard deviation units.

**Table 5:** Correlations between selected nutritional status indices.

Nutrtional Status Indices		Age	BMI	FMI	VFL	BMD T-Score
BMI	Rho	0.097				
	*p*	0.207				
FMI	Rho	0.058	0.931 **			
	*p*	0.456	<0.001			
VFL	Rho	0.334 **	0.899 **	0.944 **		
	*p*	<0.001	<0.001	<0.001		
BMD T-score	Rho	−0.087	0.345 **	0.254 **	0.222 **	
	*p*	0.258	<0.001	0.001	0.004	
MMI	Rho	0.096	0.87 **	0.650 **	0.648 **	0.419 **
	*p*	0.216	<0.001	<0.001	<0.001	<0.001

Spearman’s correlation; ** *p* < 0.01. BMI—body mass index; FMI—fat mass index; VFL—visceral fat level; BMD T-score—bone mineral density expressed in relation to a reference population in standard deviation units; MMI—muscle mass index.

**Table 6:** Nutritional status indices and physical activity by dietary pattern groups.

Characteristics	Dietary Pattern 1		Dietary Pattern 2		*p*
	X ± SD	Me	X ± SD	Me	
BMI [kg/m^2^]	25.4 ± 2.9	24.7	27.0 ± 3.2	27.0	0.001 **
FMI [kg/m^2^]	4.6 ± 1.6	4.5	5.8 ± 2.0	5.8	<0.001 **
VFL	6.03 ± 2.58	6.00	7.85 ± 2.87	8.00	<0.001 **
BMD T-score	−0.11 ± 0.85	−0.15	0.10 ± 1.00	0.05	0.214
MMI [kg/m^2^]	19.7 ± 1.5	19.3	20.1 ± 1.4	20.1	0.027 *
Physical activity (MET-minutes/week):					
total	22,554 ± 16,079	19,638	15,704 ± 13,149	12417	0.007 **
job-related physical activity	8815 ± 10,529	5667	6212 ± 7683	2925	0.215
transportation	4087 ± 4033	2772	3232 ± 3726	2014	0.249
housework, house maintenance, caring for family	5320 ± 5626	4020	3335 ± 3856	2170	0.030 *
recreation, sport and leisure-time physical activity	4331 ± 5087	2573	2926 ± 4359	1587	0.056
walking	7916 ± 6967	6584	6161 ± 6559	3663	0.201
moderate	9104 ± 7636	7410	5373 ± 5338	3540	0.002 **
intensive	5534 ± 6409	4560	4169 ± 5514	1920	0.188
Time spent sitting (h/d):					
weekdays	4.4 ± 2.3	4.5	5.0 ± 3.0	4.0	0.483
weekend	3.9 ± 1.9	4.0	4.0 ± 2.4	4.0	0.678

U Mann–Whitney Test * *p* < 0.05; ** *p* < 0.01. X—average; SD—standard deviation; Me—median; BMI—body mass index; FMI—fat mass index; VFL—visceral fat level; BMD T-score—bone mineral density expressed in relation to a reference population in standard deviation units; MMI—muscle mass index.

**Table 7:** Relationships between physical activity (IPAQ) and body mass index (BMI), fat mass index (FMI), visceral fat level (VFL) and bone mineral density (BMD T-score).

Physical Activity	BMI		FMI		VFL		BMD T-Score	
	Rho	*p*	Rho	*p*	Rho	*p*	Rho	*p*
Physical activity [MET-minutes/week]:								
total	−0.100	0.198	−0.122	0.114	−0.123	0.112	−0.048	0.534
job-related physical activity	−0.030	0.697	−0.014	0.861	−0.058	0.456	0.020	0.794
transportation	−0.080	0.303	−0.042	0.584	−0.024	0.761	−0.063	0.418
housework, house maintenance and caring for family	0.007	0.927	−0.020	0.792	0.014	0.859	−0.019	0.806
recreation, sport and leisure-time	−0.182 *	0.018	−0.277 **	<0.001	−0.228 **	0.003	−0.108	0.161
walking	−0.156 *	0.043	−0.117	0.131	−0.132	0.087	−0.140	0.070
moderate	−0.028	0.722	−0.086	0.266	−0.031	0.685	−0.006	0.941
intensive	−0.073	0.343	−0.122	0.113	−0.140	0.070	0.043	0.578
Time spent sitting [h/d]:								
weekdays	0.051	0.532	0.084	0.299	0.062	0.449	0.050	0.536
weekend	0.147	0.070	0.198 *	0.014	0.172 *	0.034	−0.090	0.270

Spearman’s correlation; * *p* < 0.05; ** *p* < 0.01. BMI—body mass index; FMI—fat mass index; VFL—visceral fat level; BMD T-score—bone mineral density expressed in relation to a reference population in standard deviation units.

**Table 8:** Logistic regression model for excess fat mass index probability (*p* < 0.001).

Variable	B	Standard Error	*p*	OR	95% Cl
Dietary pattern	1.093	0.441	0.013	2.98	1.26–7.08
IPAQ—sport and recreation	−0.560	0.240	0.020	0.57	0.36–0.91
Constant	−2.446	0.823	0.003		

B—regression coefficient; Dietary pattern: 1—more healthy, 2—less healthy.

## Data Availability

The data presented in this study are available on request from the corresponding author. The data are not publicly available due to privacy/ethical restrictions.

## Supplementary Materials

The following supporting information can be downloaded at: https://www.mdpi.com/article/10.3390/ijerph19095305/s1, Table S1: Food consumption frequency; Table S2: Relationships between diet (FFQ) and body mass index (BMI), fat mass index (FMI), visceral fat level (V Fat L), and bone mineral density (BMD T-score).
