# Supplementary material for: Association between Diet, Physical Activity and Nutritional Status of Male Border Guard Officers
Source: Int J Environ Res Public Health. 2022 Apr 27;19(9):5305. doi: 10.3390/ijerph19095305 (PMC9101595; doi:10.3390/ijerph19095305)
Supplement: Supplementary file 1 [file ijerph-19-05305-s001.zip › ijerph-1674837-supplementary.pdf]

Table S1. Food consumption frequency.

Food groups	Percentage of consumption frequency*							
	1	2	3	4	5	6	7	8
Fruits, vegetables, and potatoes								
Fruits together—all types	0	1	1	11	6	51	27	3
Stone fruits	1	5	14	29	23	24	3	1
Kiwi fruit and citrus	1	5	16	31	23	15	8	1
Other tropical fruits	5	17	25	30	15	6	1	1
Bananas	2	1	10	22	22	32	10	1
Apples and pears	1	0	5	19	14	52	8	1
Avocado	38	25	20	7	5	5	0	0
Olives	27	16	27	16	6	7	1	0
Dried fruits	10	22	22	23	11	8	4	0
Sweet fruit preserves and candied fruits	8	21	26	22	11	10	2	0
Vegetables—all types	0	0	2	10	7	49	29	3
Crucifers	1	1	8	32	25	30	2	1
Yellow–orange vegetables	0	1	2	19	31	43	3	1
Leafy green vegetables	0	2	9	29	29	27	4	0
Tomatoes	2	1	1	11	12	51	21	1
Vegetables: fresh cucumbers, squash, zucchini, pumpkin, eggplant	1	2	2	18	26	42	8	1
Root vegetables and others	1	1	6	23	23	42	4	0
Potatoes in various forms	1	2	7	15	11	55	8	1
Seeds of legumes								
Fresh seeds of legumes and canned ones	4	7	25	38	17	9	0	0
Dry seeds of legumes	7	17	34	32	8	2	0	0
Cereal products								
Wholemeal or with grains, so-called dark bread	1	4	7	15	11	36	22	4
Refined bread, so-called white bread	2	4	4	12	12	33	27	6
Unrefined groats coarse	0	5	15	27	21	30	2	0
Refined cereal grain	2	4	14	28	24	27	1	0
Ready-to-eat breakfast cereal products	15	19	15	18	14	15	4	0
Dairy products and eggs								
Milk and milk drinks	3	2	6	17	13	38	17	4
Sweetened milk drinks	8	8	17	20	15	26	5	1
Cottage cheese	1	2	6	23	20	40	8	0
Flavored cottage cheese	20	13	20	21	13	11	2	0
Cheese	2	3	9	17	14	42	12	1
Eggs and egg dishes	1	2	2	16	26	47	6	0
Meat products and fish								
Sausages, different types	0	2	6	19	14	43	15	1
High-quality cold cuts	0	1	4	12	11	51	21	0

Sausage products and offal	9	14	20	28	14	13	2	0
Red meat	1	6	12	37	19	22	2	1
Poultry and rabbit	1	1	3	24	19	48	4	0
Wild game meat	41	31	14	8	4	2	0	0
Lean fish	4	9	25	31	26	5	0	0
Oily fish	5	14	27	29	18	7	0	0
Fats, nuts and grains								
Oil, all kinds	4	1	8	20	13	48	6	0
Butter, all types	5	2	4	13	12	27	33	4
Margarine, all types	32	13	11	11	8	14	9	2
Cream, sweet or sour cream, for food or beverages	11	9	14	25	20	18	2	1
Other animal fats	29	20	19	21	6	4	1	0
Mayonnaise and dressings, i.e., salad dressings—all types	7	12	21	36	12	10	1	1
Nuts	3	8	19	30	19	18	3	0
Grains	11	17	24	25	13	8	2	0
Sweets and snacks								
Sugar to sweeten beverages	31	3	5	10	5	10	24	12
Honey to sweeten food and beverages	20	16	14	19	11	15	5	0
Chocolate, chocolate candies, and candy bars	3	5	13	24	15	34	5	1
Non-chocolate candies	15	20	23	19	12	8	2	1
Biscuits and cakes	2	12	21	24	17	23	1	0
Ice cream and pudding	3	23	32	24	13	5	0	0
Salty snacks	11	13	24	35	9	6	2	0
Soft drinks								
Fruit juices and fruit nectars	2	5	14	27	17	28	6	1
Vegetable juices and vegetable-fruit ones	10	18	20	19	18	14	0	1
Energy drinks	53	15	14	10	3	2	2	1
Sweetened sodas such as Fanta, Coca-Cola, Mirinda, Sprite	19	14	19	25	8	13	2	0
Alcoholic beverages								
Beer	3	7	11	30	21	24	4	0
Wine and drinks	9	23	23	25	14	5	1	0
Vodka and spirits	12	20	32	21	11	4	0	0

* Consumption frequency categories: 1—never or almost never; 2—once a quarter or less often; 3—once a month or less often; 4—a few times a month; 5—once a week; 6—several times a week; 7—every day; 8—several times a day.

Table S2. Relationships between diet (FFQ) and body mass index (BMI), fat mass index (FMI), visceral fat level (VFL), and bone mineral density (BMD T-score).

**Groups of products**	**BMI**		**FMI**		**VFL**		**BMD T-score**	
	**Rho**	**p**	**Rho**	**p**	**Rho**	**p**	**Rho**	**p**
**Food groups**								
Fruits, vegetables, and potatoes	−0.234**	0.002	−0.294**	<0.001	−0.216**	0.005	−0.160*	0,038
Seeds of legumes	−0.190*	0.014	−0.214**	0.005	−0.186*	0.016	−0.060	0.437
Cereal products	−0.084	0.277	−0.112	0.148	−0.135	0.081	−0.124	0.107
Dairy products and eggs	−0.228**	0.003	−0.243**	0.001	−0.234**	0.002	−0.257**	0.001
Meat products and fish	0.028	0.717	0.015	0.843	0.037	0.633	−0.049	0.526
Fats, nuts, and grains	−0.157*	0.042	−0.151	0.050	−0.129	0.095	−0.066	0.394
Sweets and snacks	−0.098	0.203	−0.051	0.513	−0.048	0.534	0.002	0.977
Non-alcoholic beverages	−0.068	0.380	−0.059	0.446	−0.103	0.182	−0.087	0.259
Alcoholic beverages	0.076	0.326	0.075	0.336	0.060	0.439	0.056	0.471
**Selected products**								
Fruits, vegetables, and potatoes								
Fruits together - all types	−0.175*	0.026	−0.210**	0.008	−0.152	0.054	−0.050	0.527
Stone fruits	−0.125	0.106	−0.154*	0.046	−0.054	0.490	−0.120	0.121
Kiwi fruit and citrus	−0.126	0.103	−0.172*	0.025	−0.128	0.098	−0.039	0.616
Other tropical fruits	−0.276**	<0.001	−0.346**	<0.001	−0.276**	<0.001	−0.133	0.089
Bananas	−0.253**	0.001	−0.280**	<0.001	−0.264**	0.001	−0.080	0.303
Apples and pears	−0.087	0.262	−0.124	0.109	−0.059	0.450	−0.052	0.505
Avocado	−0.030	0.705	−0.092	0.243	−0.085	0.282	−0.023	0.769
Olives	−0.055	0.479	−0.092	0.240	−0.023	0.770	−0.095	0.225
Dried fruits	−0.137	0.077	−0.248**	0.001	−0.185*	0.017	−0.058	0.457
Sweet fruit preserves and candied fruits	−0.152*	0.049	−0.200**	0.009	−0.207**	0.007	−0.014	0.858
Vegetables - all types	−0.208**	0.009	−0.251**	0.002	−0.227**	0.004	−0.116	0.149
Crucifers	−0.124	0.110	−0.190*	0.014	−0.114	0.141	−0.037	0.631
Yellow–orange vegetables	−0.181*	0.019	−0.215**	0.005	−0.172*	0.026	−0.218**	0.004
Leafy Green vegetables	−0.217**	0.005	−0.245**	0.001	−0.160*	0.039	−0.133	0.085
Tomatoes	−0.102	0.187	−0.175*	0.024	−0.106	0.171	−0.167*	0.030
Vegetables: fresh cucumbers, squash, zucchini, pumpkin, eggplant	−0.126	0.103	−0.172*	0.025	−0.124	0.108	−0.060	0.437
Root vegetables and others	−0.076	0.324	−0.100	0.194	−0.056	0.471	−0.123	0.112
Potatoes in various forms	−0.207**	0.007	−0.173*	0.025	−0.137	0.076	−0.167*	0.030
Seeds of legumes								
Fresh seeds of legumes and canned ones	−0.171*	0.027	−0.179*	0.020	−0.166*	0.031	−0.059	0.447
Dry seeds of legumes	−0.120	0.122	−0.169*	0.028	−0.145	0.060	0.006	0.943
Cereal products								
Wholemeal or with grains, so-called dark bread	−0.017	0.831	−0.105	0.175	−0.057	0.460	−0.041	0.595
Refined bread, so-called white bread	0.007	0.932	0.064	0.406	0.055	0.476	−0.061	0.429
Unrefined groats coarse	−0.163*	0.034	−0.188*	0.015	−0.219**	0.004	−0.112	0.147

Refined cereal grain	−0.168*	0.029	−0.163*	0.034	−0.210**	0.006	−0.142	0.066
Ready-to-eat breakfast cereal products	−0.173*	0.025	−0.199**	0.009	−0.225**	0.003	−0.037	0.634
Dairy products and eggs								
Milk and milk drinks	−0.203**	0.008	−0.225**	0.003	−0.217**	0.005	−0.145	0.059
Sweetened milk drinks	−0.125	0.105	−0.065	0.406	−0.098	0.207	−0.109	0.161
Cottage cheese	−0.108	0.163	−0.135	0.081	−0.113	0.144	−0.204**	0.008
Flavored cottage cheese	0.033	0.676	0.048	0.536	0.059	0.447	0.009	0.907
Cheese	−0.106	0.171	−0.090	0.245	−0.065	0.400	−0.139	0.072
Eggs and egg dishes	−0.134	0.083	−0.177*	0.021	−0.172*	0.026	−0.240**	0.002
Meat products and fish								
Sausages, different types	0.116	0.133	0.124	0.108	0.141	0.068	−0.034	0.660
High-quality cold cuts	0.069	0.375	0.055	0.480	0.093	0.228	−0.005	0.948
Sausage products and offal	−0.004	0.963	−0.025	0.752	0.010	0.901	−0.017	0.826
Red meat	0.024	0.756	0.045	0.565	0.034	0.664	−0.043	0.579
Poultry and rabbit	−0.133	0.086	−0.114	0.143	−0.134	0.084	−0.103	0.185
Wild game meat	−0.056	0.467	−0.096	0.214	−0.113	0.145	0.105	0.173
Lean fish	−0.139	0.072	−0.164*	0.033	−0.109	0.159	−0.056	0.468
Oily fish	−0.056	0.472	−0.103	0.183	−0.052	0.503	−0.053	0.495
Fats, nuts and grains								
Oil, all kinds	−0.128	0.098	−0.113	0.145	−0.127	0.102	−0.043	0.583
Butter, all types	−0.186*	0.017	−0.121	0.122	−0.110	0.162	−0.029	0.713
Margarine, all types	0.079	0.309	0.094	0.225	0.088	0.256	−0.016	0.842
Cream, sweet or sour cream, for food or beverages	0.025	0.747	0.048	0.538	0.029	0.708	−0.003	0.969
Other animal fats	−0.037	0.633	−0.014	0.852	−0.066	0.391	−0.004	0.964
Mayonnaise and dressings, i. .e., salad dressings - all types	−0.008	0.913	0.028	0.718	0.011	0.882	−0.049	0.525
Nuts	−0.244**	0.001	−0.350**	<0.001	−0.306**	<0.001	0.022	0.780
Grains	−0.159*	0.040	−0.233**	0.002	−0.188*	0.015	−0.003	0.965
Sweets and snacks								
Sugar to sweeten beverages	−0.003	0.970	0.057	0.468	0.075	0.338	0.065	0.407
Honey to sweeten food and beverages	−0.164*	0.037	−0.233**	0.003	−0.217**	0.005	−0.183*	0.019
Chocolate, chocolate candies, and candy bars	−0.084	0.280	−0.031	0.692	−0.045	0.565	−0.086	0.267
Non-chocolate candies	−0.055	0.482	−0.031	0.689	−0.050	0.523	0.068	0.387
Biscuits and cakes	−0.113	0.144	−0.039	0.619	−0.050	0.517	0.016	0.832
Ice cream and pudding	−0.033	0.675	0.012	0.880	0.021	0.782	−0.009	0.906
Salty snacks	−0.036	0.638	0.069	0.375	−0.027	0.728	0.010	0.901
Soft drinks								
Fruit juices and fruit nectars	−0.069	0.377	−0.082	0.293	−0.094	0.226	0.017	0.829
Vegetable juices and vegetable-fruit ones	−0.153*	0.047	−0.214**	0.005	−0.170*	0.027	−0.133	0.086
Energy drinks	0.004	0.961	0.005	0.953	−0.097	0.211	−0.028	0.720
Sweetened sodas such as Fanta, Coca-Cola, Mirinda, Sprite	0.070	0.364	0.160*	0.037	0.081	0.296	−0.022	0.779
Alcoholic beverages								

Beer	0.051	0.513	0.046	0.558	0.019	0.806	−0.031	0.686
Wine and drinks	−0.017	0.832	−0.021	0.792	−0.007	0.923	0.013	0.863
Vodka and spirits	0.081	0.293	0.091	0.239	0.078	0.315	0.130	0.092

Spearman's correlation; * $p < 0.05$; ** $p < 0.01$.
